# Supplementary material for: Optimization of Mature Embryo-Based Tissue Culture and Agrobacterium-Mediated Transformation in Model Grass Brachypodium distachyon
Source: Int J Mol Sci. 2019 Oct 31;20(21):5448. doi: 10.3390/ijms20215448 (PMC6862288; doi:10.3390/ijms20215448)
Supplement: Supplementary file 1 [file ijms-20-05448-s001.pdf]

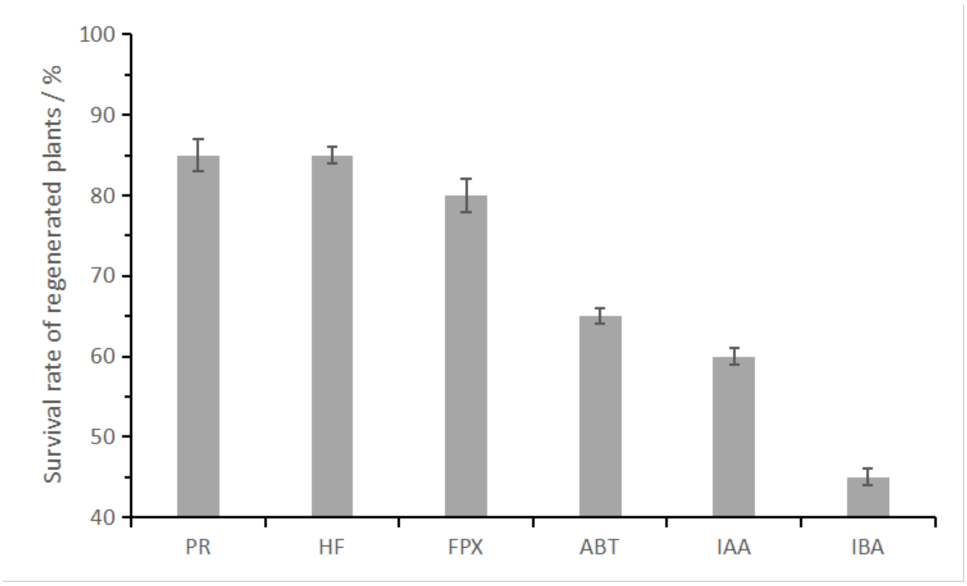

Supplementary Figure 1. Survival rate of regenerated plants between PR and RIM-based methods. For each treatment, three replicates were conducted. Error bars represent ± SE, n=3. HF, hormone-free RIM. ABT, ABT-2 rooting powder. IAA, 3-Indole acetic acid. IBA, Indole-4-butyric acid.
